# Molecular characterisation and expression analysis of two heat-shock proteins in *Taenia multiceps*

**DOI:** 10.1186/s13071-019-3352-8

**Published:** 2019-03-12

**Authors:** Yuchen Liu, Cheng Guo, Xiaowei Dong, Xiaobin Gu, Yue Xie, Weimin Lai, Xuerong Peng, Guangyou Yang

**Affiliations:** 1 Department of Parasitology, College of Veterinary Medicine, Sichuan Agricultural University, Wenjiang, 611130 China; 2 Department of Chemistry, College of Life and Basic Science, Sichuan Agricultural University, Wenjiang, 611130 China

**Keywords:** *Taenia multiceps*, Coenurus cerebralis, Metacestode, Heat-shock protein, Immunohistochemistry, Quantitative real-time PCR, Indirect ELISA

## Abstract

**Background:**

*Taenia multiceps* is a harmful tapeworm and its larval form (coenurus cerebralis) is the causative agent of coenurosis, a disease affecting the health of herbivores, resulting in great economic loss to animal husbandry. Heat-shock proteins (HSPs), expressed in all prokaryotes and eukaryotes, act as molecular chaperones and can affect pathogenicity.

**Methods:**

Herein, cDNAs of *T. multiceps* genes *Tm*-HSP60 and *Tm*-p36 were cloned and molecularly characterised by bioinformatics analyses. The immunogenicity and immunoreactivity of recombinant r*Tm*-HSP60 and r*Tm*-p36 proteins were investigated by immunoblotting and indirect ELISA was established to evaluate their serodiagnostic potential. Tissue localisation and transcriptional level at different life stages of *T. multiceps* were determined by immunohistochemical and quantitative real-time PCR analyses.

**Result:**

The 533 residue r*Tm*-HSP60 and the 314 residue r*Tm*-p36 proteins share typical highly conserved features of HSPs. *Tm*-p36 shares structural characteristics with metazoan small HSPs, with two N-terminal α-crystallin domains. Compared with *Tm*-p36, *Tm*-HSP60 displayed stronger immunogenicity, and the indirect ELISA based on r*Tm*-HSP60 exhibited a sensitivity of 83.3% and a specificity of 87.5%, while r*Tm*-p36 was not suitable to develop indirect ELISA. *Tm*-HSP60 was widely distributed in all stages of *T. multiceps*, albeit at relatively low levels, while *Tm*-p36 was specifically distributed in the protoscolex and oncosphere.

**Conclusions:**

The sequence, structural and functional analyses of these two HSPs indicates that they may play important roles in the life-cycle of *T. multiceps* as molecular chaperones. *Tm*-HSP60 displayed stronger immunogenicity compare to *Tm*-p36, and has the potential for antibody detection. *Tm*-p36 was strongly associated with the activation of oncospheres and has potential interest for vaccination.

**Electronic supplementary material:**

The online version of this article (10.1186/s13071-019-3352-8) contains supplementary material, which is available to authorized users.

## Background

*Taenia multiceps* is a common taeniid cestode and multi-host parasite. Adult worms adhere to the small intestine of canids, while the larval stage (coenurus cerebralis) causes coenurosis, a disease affecting herbivores, resulting in great economic loss to animal husbandry in Europe, the USA, Africa and Asia [1–4]. The larval parasites are localized in the central nervous system, as well as subcutaneous and muscle tissue of the intermediate host, most of which are herbivorous animals [5–7], but humans can also act as intermediate hosts if eggs are eaten by mistake [8].

*Taenia multiceps* has a complex life-cycle that involves different phases of development and migration within the host [9, 10]. To overcome possible obstacles, parasites have developed tolerance mechanisms to assist survival and proliferation. One mechanism counteracts stress *via* the expression of heat-shock proteins (HSPs) that act as important molecular chaperones [11]. HSPs ensure the correct folding, processing and functionality of their protein substrates. In addition to facilitating cytoprotection against stress, HSPs also participate in the differentiation and development of parasites, and may even serve as diagnostic or vaccine candidates [12].

HSPs in some cestodes have been characterised, such as HSP60 and HSP70 that are immunodominant antigens in *Echinococcus granulosus* and *T. multiceps* [13, 14], and p36, a unique type of small HSP (sHSP) in metazoans such as *Taenia saginata*, *Taenia solium* and *E. granulosus* [15–18]. However, previous research on HSPs in cestodes is limited to exploring immunogenicity, and the relevance to biological functions throughout other life-cycle stages is unknown.

Herein, we investigated HSP60 and p36 from *T. multiceps*, identified previously from transcriptomic analyses [19, 20]. We expressed the recombinant proteins using a prokaryotic system, and performed bioinformatics analyses, tissue localisation studies, and analysed their transcriptional level by quantitative real-time PCR (qPCR) to probe their possible biological functions. This is a preliminary exploration of the roles of these two proteins in *T. multiceps*, and the findings lay the foundation for further understanding of their functions.

## Methods

### Parasites and animals

Cysts of *T. multiceps* were obtained from naturally infected goats in Sichuan Province, China. Adult worms were obtained from intestine samples from four 2-month-old dogs at 30 days after infection with 20,000 protoscolices. Mature eggs were obtained from fresh gravid proglottids, and oncospheres were released from the proglottids using trypsin and used for RNA extraction. Nine-week-old female New Zealand white rabbits were obtained from Dashuo Experimental Animal Co., Ltd (Chendu, China).

### Primers

The cDNA sequences of *Tm*-HSP60 and *Tm*-p36 were amplified using primers designed from Unigene 5021 and 18510 of the assembled *T. multiceps* transcriptome dataset, which is homologous to the genome sequences of *Echinococcus multilocularis* (GenBank: CDS35950.1) and *T. saginata* (GenBank: Q7YZT0.1). All primers (Table 1) were synthesised by Sangon (Shanghai, China).

**Table 1:** Primers used for amplification and qPCR. All primers used for amplification included *BamH*I or *EcoR*I restriction enzyme sites (underlined)

Primer	Sequence (5′–3′)
*Tm*-HSP60F^a^	CGGGATCCATGCTTGTTGGTGTCGAT
*Tm*-HSP60R^a^	CGGAATTCTCACATCATACCGCCC
*Tm*-p36F^a^	CGGGATCCATGTCCATCTTTCCG
*Tm*-p36R^a^	CGGAATTCTCATTTAAAGAGAGGCG
*Tm*-HSP60F^b^	TGCGTGATATGGCTATTGCGTCTG
*Tm*-HSP60R^b^	GAGCGTGTCGTCCTTGGTGATG
*Tm*-p36F^b^	CGAGAGTGTGATGAAGGAGATGAGTG
*Tm*-p36R^b^	ACTTGTTCTTGTCCGCCTTGATGG
actinF	CTAAGGCGAACCGTGAGAAGATGAC
actinR	GGCATGAGGCAAGGCGTAACC

^a^For amplification

^b^For qPCR

### Bioinformatics analyses

Bioinformatics analyses were performed using ORF Finder (http://www.ncbi.nlm.nih.gov/gorf/gorf.html) to predict open reading frames (ORFs), the TMHMM Sever 2.0 (http://www.cbs.dtu.dk/services/TMHMM-2.0) to identify transmembrane regions, and half-life and instability indices were predicted with the ExPASy Proteomics Server (http://web.Expasy.org/protparam/). Signal peptides were predicted using the SignalP server (http://www.cbs.dtu.dk/Services/SignalP/), subcellular localisation and B-cell epitopes were predicted by BaCelLo (http://gpcr.biocomp.unibo.it/bacello/pred.htm) and Antibody Epitope Prediction (http://tools.immuneepitope.org/bcell/), and protein tertiary structures were predicted using SWISS-MODEL (http://swissmodel.expasy.org/). Multiple sequence alignment was performed with Clustal X software version 1.83.

### Cloning, expression and purification of recombinant proteins

TRIzol reagent (Tiangen, Beijing, China) was used to extract total RNA from adult worms, protoscolices and oncospheres. cDNA was reverse-transcribed using a RevertAid First Strand cDNA Synthesis Kit following the manufacturer’s instructions (Thermo Fisher Scientific, Vilnius, Lithuania). Target fragments were amplified, ligated into the pET32a(+) vector (TaKaRa, Dalian, China) and transformed into BL21 (DE3) competent cells. Expression of the recombinant proteins was induced by 1 mM isopropyl β-d-1-thiogalactopyranoside (IPTG) and purification was achieved using Ni^2+^ affinity chromatography (Bio-Rad, Hercules, CA, USA).

### Sera

Corresponding sera of *T. multiceps* (24 samples), *Taenia hydatigena* (12 samples), *Fasciola hepatica* (12 samples) and *Haemonchus contortus* (12 samples) were isolated from naturally infected goats in Sichuan Province and sera of *E. granulosus* (12 samples), *Moniezia expansa* (12 samples) were isolated from naturally infected sheep. Serum samples from cestode-free goats at autopsy were used as negative control.

Four rabbits were used to prepare polyclonal antibodies of *Tm*-HSP60 and *Tm*-p36, and rabbit serum samples were obtained before immunisation as a negative control. The first immunisation was a subcutaneous injection with 100 μg recombinant protein mixed with same volume of Freund’s complete adjuvant (Sigma-Aldrich, St Louis, MO, USA). The second and third immunisations were injections with 100 μg recombinant protein mixed with same volume of Freund’s incomplete adjuvant. The immunisation interval was 1 week. Two weeks after the final immunisation, serum samples were collected and the serum titre was determined by enzyme-linked immunosorbent assay (ELISA). Immunoglobulin G (IgG) was further isolated from the serum using a Protein G-Sepharose column (Bio-Rad, Richmond, USA).

### Western blotting analysis

A Mammalian Protein Extraction Kit (Solarbio, Beijing, China) was used to extract crude proteins from adult worms. Proteins were separated by 12% sodium dodecyl sulphate-polyacrylamide gel electrophoresis (SDS-PAGE) and transferred to a nitrocellulose membrane using an electrophoretic transfer cell (Bio-Rad, Hercules) for 30 min. After washing with TRIS-buffered saline containing Tween-20 (TBST), membranes were incubated with 5% (w/v) skimmed milk at 37 °C for 2 h and incubated at 4 °C with polyclonal antibodies (1:200 v/v dilution) or serum from infected goats (1:200 v/v dilution) for 12 h, then with a 1:1,000 dilution of horseradish peroxidase (HRP)-conjugated goat anti-rabbit IgG or rabbit anti-goat IgG (Boster, Wuhan, China) for 2 h. Signals were measured using an Enhanced HRP-DAB Chromogenic Substrate Kit (Tiangen).

### Immunolocalisation of *Tm*-HSP60 and *Tm*-p36 at different life stages of *T. multiceps*

Samples (adult worms and protoscolices) were washed with phosphate-buffered saline (PBS), fixed in 4% (w/v) paraformaldehyde, embedded, and sliced into 5 μm thick sections. After dewaxing and dehydration, sections were treated with 0.01 M citrate buffer and incubated at 37 °C with 5% (w/v) skimmed milk for 4 h, then incubated with polyclonal antibodies or negative control rabbit serum (1:200 v/v) overnight at 4 °C. After washing, sections were incubated at 37 °C with fluorescein isothiocyanate-conjugated goat anti-rabbit IgG (1:200 v/v) for 1 h in the dark and signals were monitored using a fluorescence microscope (Nikon, Tokyo, Japan).

### Transcriptional profiles of *Tm*-HSP60 and *Tm*-p36 at different life stages of *T. multiceps*

For qPCR, 20 μl reactions were performed following the manual accompanying SsoAdvanced Universal SYBR Green (Bio-Rad, Hercules) with 2 μl of cDNA obtained from different proglottid and life-cycle stages (adult worms, protoscolices and oncospheres) as templates. The program involved an initial denaturation at 95 °C for 30 s, followed by 40 cycles at 95 °C for 5 s, 56 °C for 31 s and 95 °C for 15 s, and a final extension step at 65 °C for 1 min and 95 °C for 15 s. Transcriptional levels of *Tm*-HSP60 and *Tm*-p36 were estimated by the 2^−∆∆Ct^ method and a housekeeping gene actin was used as an internal control for normalization (Table 1).

### ELISA

To evaluate the serodiagnostic potential of recombinant proteins, an indirect ELISA was performed following the standard checkerboard titration procedures [21]. Briefly, proteins were diluted into six concentration gradients (ranging from 75 to 2 μg/ml) in 0.1 M carbonate buffer (pH 9.6) and coated with 100 μl per well in polystyrene 96-well microtiter plates. After incubation at 4 °C for 12 h, plates were washed three times using phosphate-buffered saline-Tween-20 (PBST) then blocked with 5 % (w/v) skim milk at 37 °C for 1.5 h. After washing three times, plates were incubated with 100 μl serum samples diluted in PBS (concentration ranging from 1:20 to 1:640) at 37 °C for 1 h. Following washing, 100 μl of HRP-labeled rabbit anti-goat or sheep IgG (Boster) diluted 1:2000 by PBS were added and incubated at 37 °C for 1 h. The substrate TMB (Tiangen) was used to react and stopped with 2 M H_2_SO_4_. The optical density at 450 nm (OD_450_) was recorded using a microplate reader (Thermo Scientific, Pittsburgh, PA, USA). We chose the optimal working conditions which gave the highest P/N value, and the cut-off value was determined by 24 negative goat serum samples, which was calculated as the mean OD_450_ plus three standard deviations (SD).

To further investigate the feasibility of the indirect ELISA, 24 serum samples from goats infected with *T. multiceps* were evaluated and the sensitivity was calculated as ELISA positive × 100/true *T. multiceps-*positive. Meanwhile, a total of 60 serum samples (12 samples for each parasite) derived from sheep infected with *E. granulosus* and *M. expansa*, and goats infected with *T. hydatigena*, *F. hepatica* and *H. contortus* were used to evaluate the cross-reactivity. Furthermore, an additional 24 negative serum samples were evaluated to determine the specificity of the indirect ELISA: ELISA negative × 100/true *T. multiceps*-negative.

### Statistical analyses

All data are presented as the mean ± SD. Statistical analyses were performed using Student’s t-tests for comparison between life stages, and Mann-Whitney U-tests for different serum groups. All statistical tests were performed using SPSS version 20.0 (SPSS Inc., Chicago, IL, USA). *P*-values < 0.05 were considered to be significant.

## Results

### Bioinformatics analyses

The 1602 nucleotide ORF in the cDNA sequence of *Tm*-HSP60 (GenBank: MH595536) encodes a 533 amino acid (aa) polypeptide, while the 945 nucleotide ORF in the cDNA sequence of *Tm*-p36 (GenBank: MH595535) encodes a 314 residue protein. No signal peptide was predicted for either protein, and neither appears to possess transmembrane regions. The predicted subcellular localisation of *Tm*-HSP60 is mitochondria, compared with nuclei for *Tm*-p36.

**Fig. 1:**
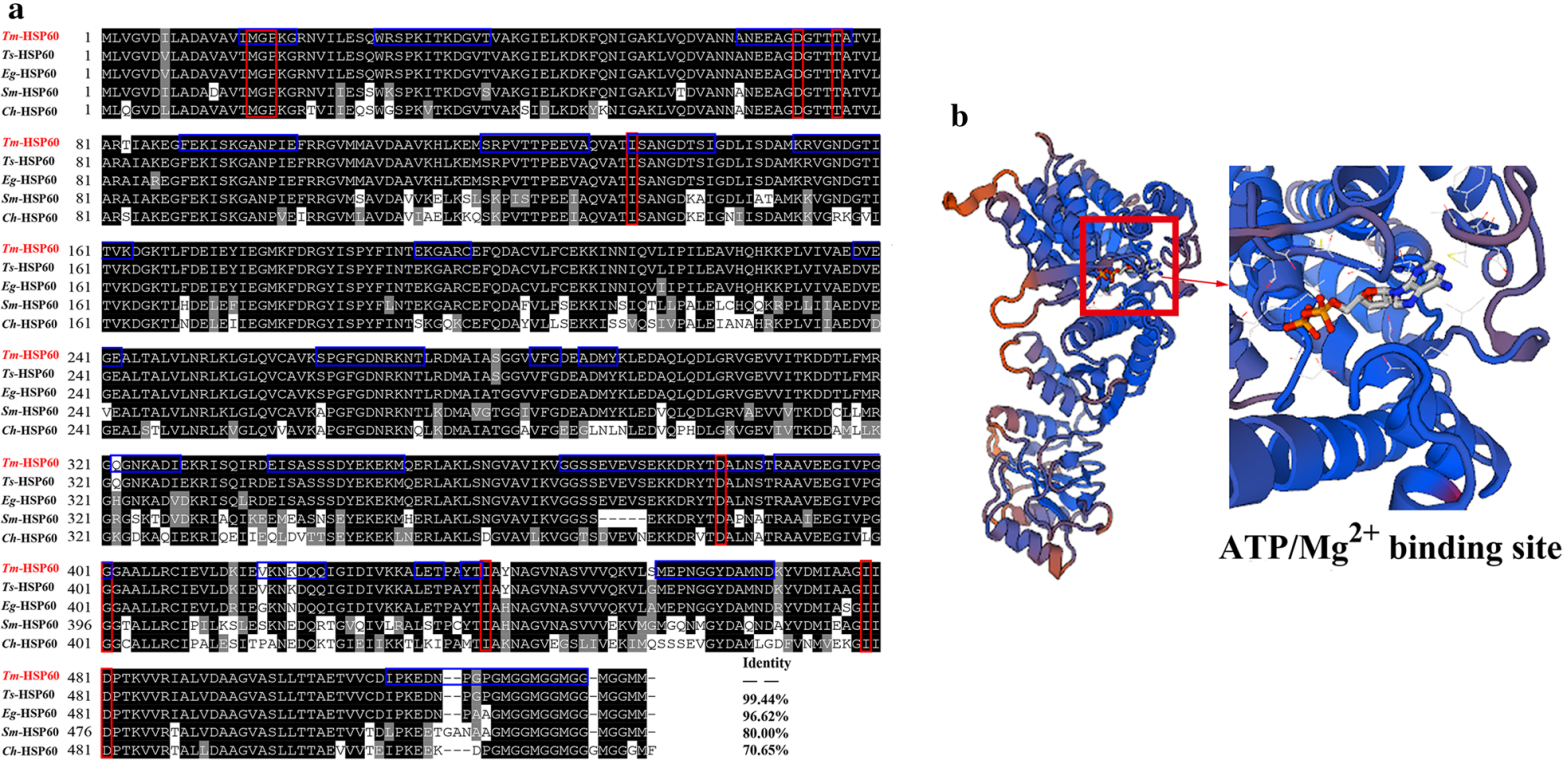
Multiple sequence alignment of *Tm*-HSP60 and three-dimensional structural model of *Tm*-HSP60. **a** Multiple sequence alignment of the deduced amino acid sequence of *Tm*-HSP60 with homologous sequences of related proteins from other parasites: *Taenia solium* (geneDB ID: TsM_000546800), *Echinococcus granulosus* (GenBank: CDS 22737.1), *Schistosoma mansoni* (GenBank: XP 018645622.1) and *Capra hircus* (GenBank: XP 017916760.1). Conserved residues are highlighted by a black background. B-cell epitopes are marked with a blue box. ATP/Mg^2+^ binding site of *Tm*-HSP60 are marked with red boxes. **b** Three-dimensional structural model of *Tm*-HSP60 and details of the ATP/Mg^2+^ binding site. The structure of *Tm*-HSP60 was modelled based on the crystal structure of *Chlorobium tepidum* HSP60 (*Ct*-HSP60, SMTL ID 5da8)

*Tm*-HSP60 is highly conserved, sharing 99.44, 96.62 and 80% sequence identity with orthologs in *T. solium* (geneDB ID: TsM_000546800), *E. granulosus* (GenBank: CDS 22737.1) and *Schistosoma mansoni* (GenBank: XP 018645622.1; Fig. 1a). *Tm*-HSP60 shares the structural and functional characteristics of the molecular chaperone superfamily and is similar to HSP60 in other organisms. The structure includes typical ATP/Mg^2+^ binding sites, protein hinge regions, and oligomerisation shared with homologous chaperone proteins (Fig. 1b).

**Fig. 2:**
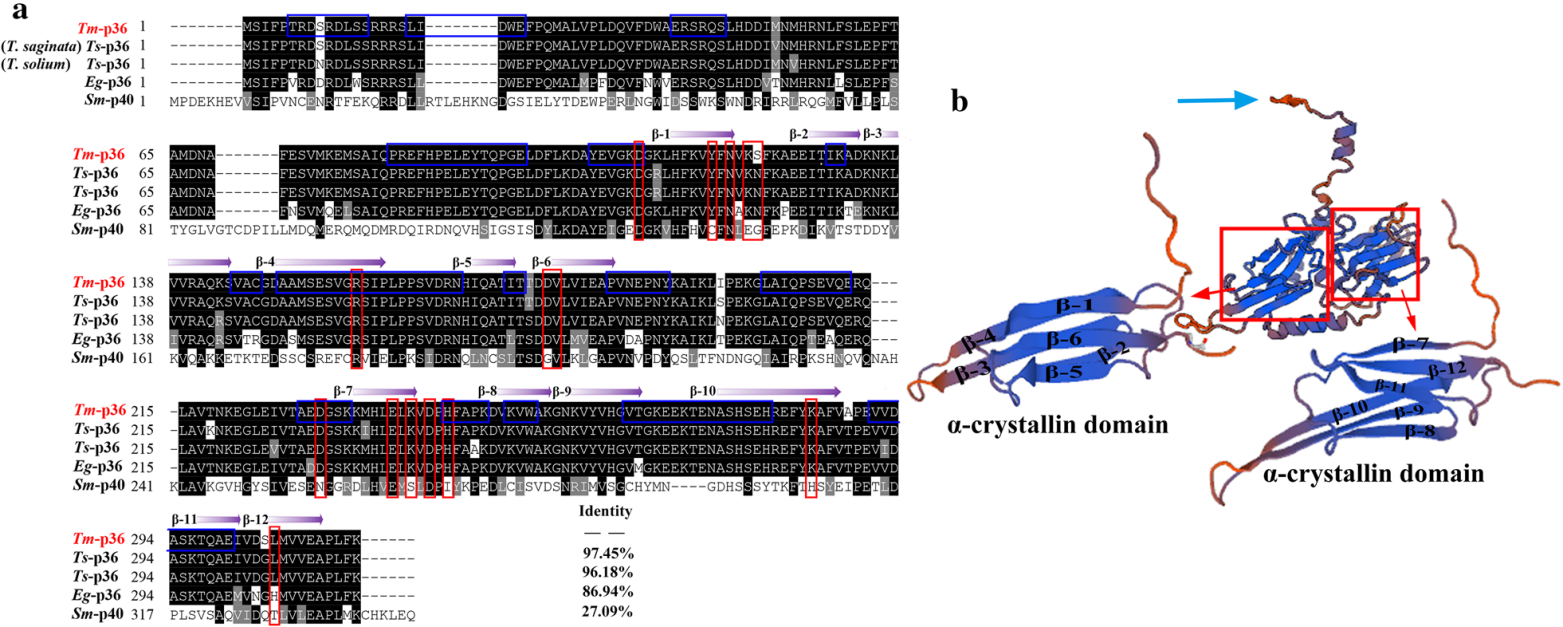
Multiple sequence alignment of *Tm*-p36 and three-dimensional structural model of *Tm*-p36. **a** Multiple sequence alignment of the deduced amino acid sequence of *Tm*-p36 with homologous sequences of related proteins from other parasites: *Taenia saginata* (GenBank: CAD80255.1), *T. solium* (GenBank: CAD36617.1), *E. granulosus* (GenBank: EUB62057.1) and *S. mansoni* (GenBank: XP 018649073.1). Conserved regions are highlighted by a black background. B-cell epitopes are marked with a blue box. Functional sites of *Tm*-p36 are marked with red boxes. β-sheets are marked with arrows. **b** Three-dimensional structural model of *Tm*-p36 and details of the N-terminal α-crystallin domain. The blue arrow indicates the C-terminus. The model of *Tm*-p36 was based on the crystal structure of *T. solium* p36 (*Ts*-p36, SMTL ID 2bol)

The *Tm*-p36 sequence is most similar to orthologs in *T. saginata* (GenBank: CAD80255.1), *T. solium* (GenBank: CAD36617.1), *E. granulosus* (GenBank: EUB62057.1) and *S. mansoni* (GenBank: XP 018649073.1), sharing 97.45, 96.18, 86.94 and 27.09% sequence identity, respectively (Fig. 2a). The tertiary structure of *Tm*-p36 was predicted to include two N-terminal α-crystallin domains composed of 12 β-sheets (Fig. 2b).

### Cloning, expression and purification of r*Tm*-HSP60 and r*Tm*-p36

**Fig. 3:**
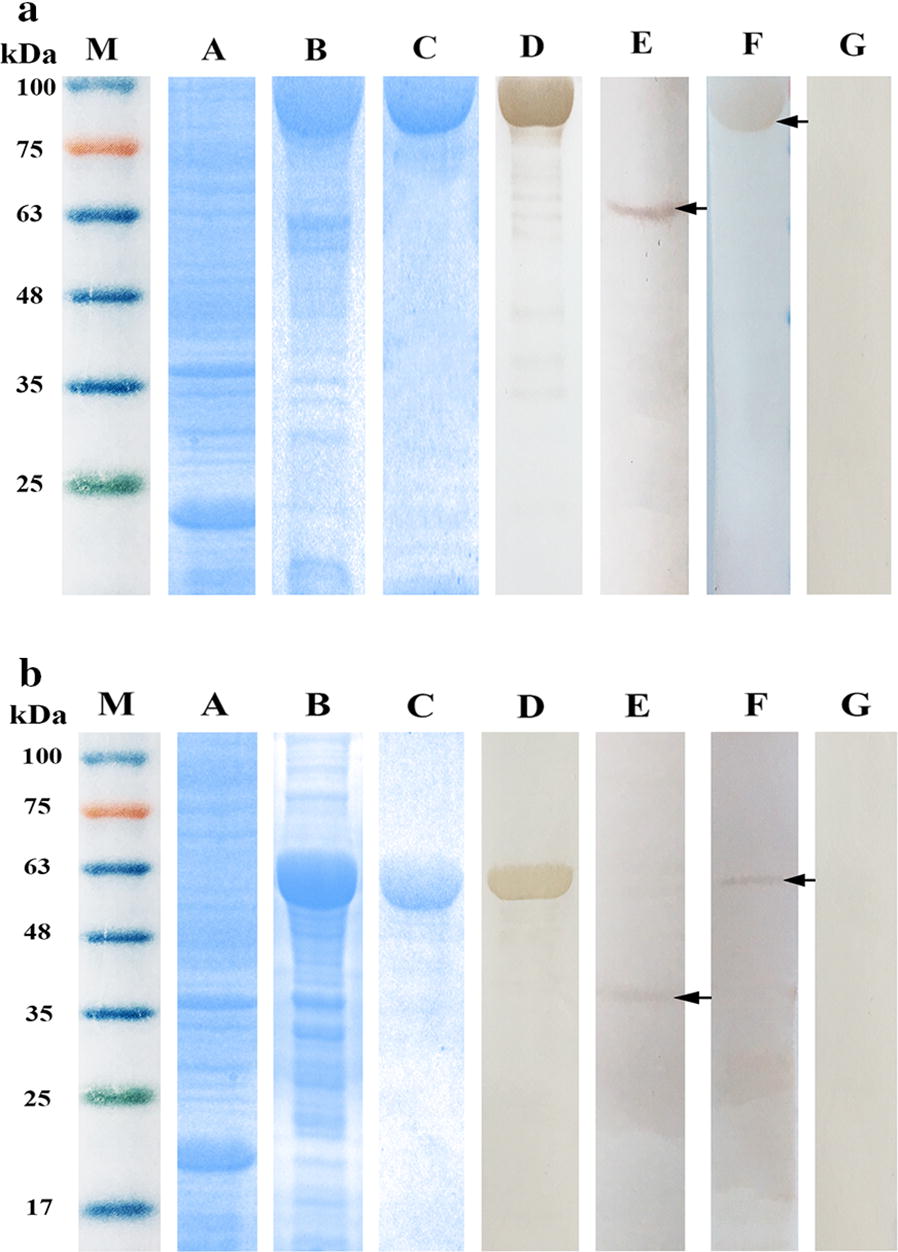
SDS-PAGE and western blot analysis of *Tm*-HSP60 (**a**) and *Tm*-p36 (**b**). Lane M: protein molecular weight markers; Lane A: crude extracts of *Escherichia coli* expressing pET32a(+) induced by IPTG; Lane B: crude extracts of *E. coli* expressing pET32a(+)-*Tm*-HSP60/*Tm*-p36 induced by IPTG; Lane C: purified recombinant proteins (10 µg); Lane D: western blot of recombinant proteins (10 µg) incubated with polyclonal antibodies; Lane E: western blot of total *T. multiceps* adult worm extract (30 µg) probed with polyclonal antibodies (arrow indicates the location of specific band); Lane F: western blot of purified recombinant proteins (10 µg) incubated with coenurus cerebralis-infected goat serum (arrow indicates the location of specific band); Lane G: negative control goat serum

*Tm*-HSP60 and *Tm*-p36 genes were amplified from adult worms, expressed in a prokaryotic expression system, and the resulting fusion proteins yielded single bands of ~76 kDa and ~54 kDa (including the ~18 kDa epitope tag fusion peptide from pET-32a) following separation by 12% SDS-PAGE (Fig. 3).

### Western blotting

The r*Tm-*HSP60 protein was reacted with polyclonal antibodies against r*Tm*-HSP60 and subjected to reaction with sera from goats infected with coenurus cerebralis; the results indicated it has some immunogenicity. The r*Tm*-p36 protein was recognised by polyclonal antibodies against r*Tm*-p36, but reaction with sera from infected goats was weak (see Additional file 1: Figure S1), and none in the negative controls. The native *Tm*-HSP60 and *Tm*-p36 proteins in adult worms were identified using polyclonal antibodies, yielding single bands of ~60 kDa and ~36 kDa (Fig. 3).

### Immunolocalisation of *Tm*-HSP60 and *Tm*-p36 at different life stages of *T. multiceps*

**Fig. 4:**
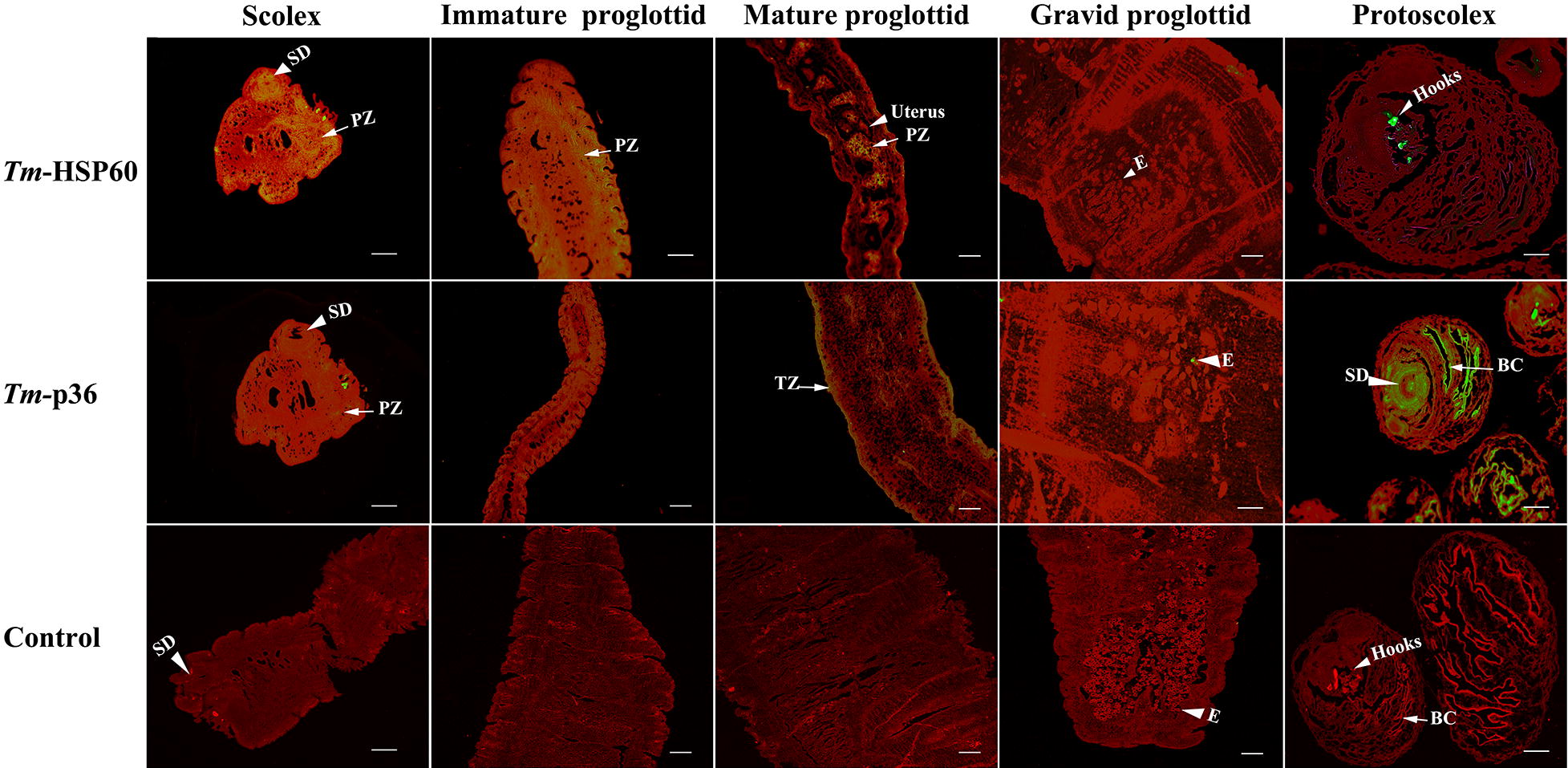
Immunohistochemical localisation of *Tm*-HSP60 and *Tm*-p36 in different proglottid tissues and different life stages of *T. multiceps*. Images of scolex, protoscolex, immature of *Tm*-HSP60 and gravid proglottid of *Tm*-p36; images of control in mature proglottids are magnified at ×400, and other images are magnified at ×200. Tissues and organs are marked with arrows. *Scale-bars*: 200 μm. *Abbreviations*: SD, sucking disc; PZ, parenchymatous zone; E, egg; TZ, tegument zone; BC, body cavity

Localisation of *Tm*-HSP60 and *Tm*-p36 in different stages of proglottid development was performed by immunofluorescence analysis using rabbit antibodies. A small amount of *Tm*-Hsp60 was found to be present in all stages of development of adult *T. multiceps*, mainly in the parenchymatous zone of the scolex, immature and mature proglottids, and also on the small hooks of the protoscolex, but there was no signal in gravid proglottids. *Tm*-p36 was found to be mainly distributed in the parenchyma and tegument of proglottids, with a small amount in the eggs of gravid proglottids, but much larger amounts in the body cavity of the protoscolex (Fig. 4).

### Transcriptional profiles of *Tm*-HSP60 and *Tm*-p36 at different stages of proglottid development and life stages of *T. multiceps*

**Fig. 5:**
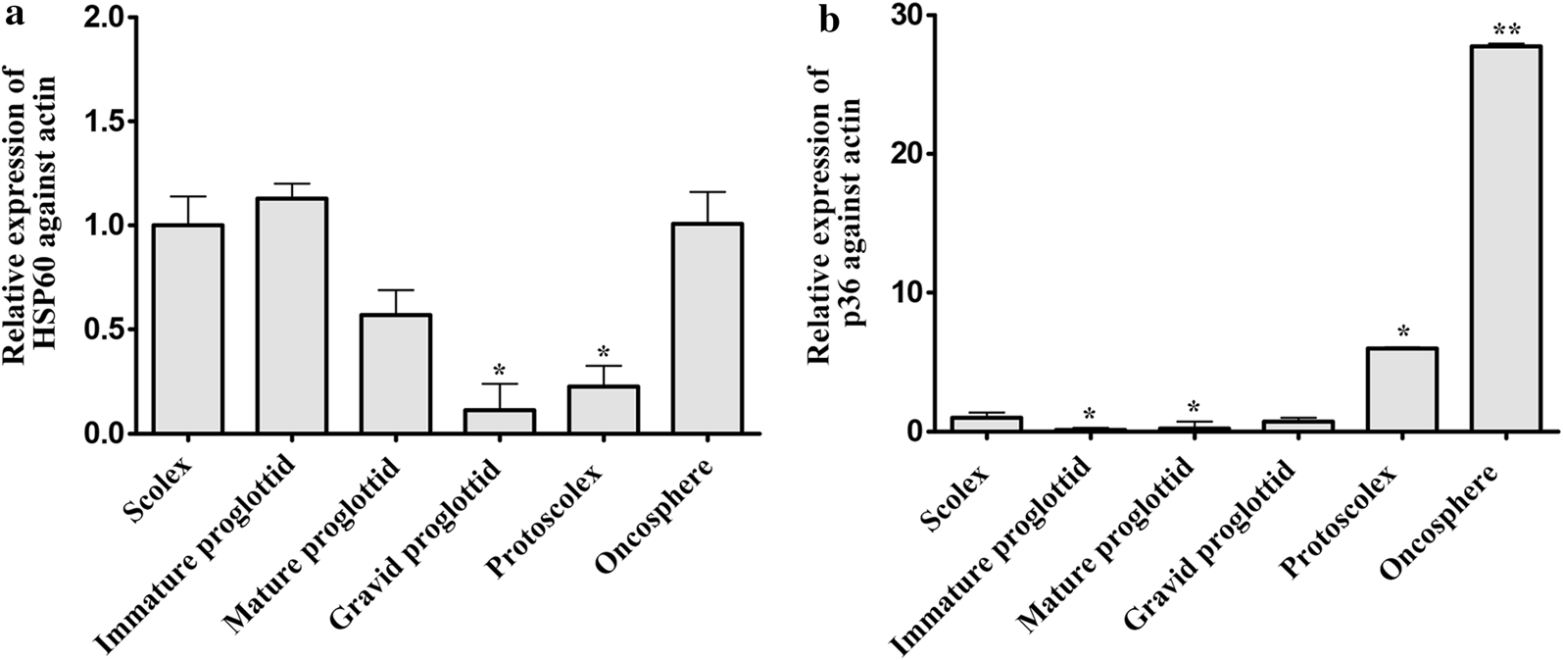
Relative expression patterns of *Tm*-HSP60 (**a**) and *Tm*-p36 (**b**). Data are presented as the mean and standard deviation (SD) of triplicate experiments. Statistically significant differences between scolex (controls) and other samples were determined using Student’s t-tests (*P < 0.05, **P < 0.01)

Transcriptional levels of *Tm*-HSP60 in different proglottid tissues were ordered immature proglottid > scolex > mature proglottid > gravid proglottid. Transcriptional profiles of *Tm*-HSP60 at different life stages were ordered oncosphere > protoscolex (Fig. 5a). By comparison, the levels of *Tm*-p36 in different proglottid tissues were ordered scolex > gravid proglottid > mature proglottid > immature proglottid, and at different life stages the order was oncosphere > protoscolex. Interestingly, the relative transcriptional level of *Tm*-p36 in oncosphere was consistently higher than in other life stages (Fig. 5b).

### Serodiagnostic potential of r*Tm*-HSP60

The r*Tm*-HSP60 has definite potential for serological diagnosis through the established indirect ELISA. From standard checkerboard titration procedures, the optimum coating concentration of r*Tm*-HSP60 was found to be 0.2 μg/well and the optimum dilution ratio of serum was 1:160, with the highest P/N value of 2.255 (see Additional file 2: Table S1). Under optimal conditions, the average OD_450_ value of 24 negative serum samples was 0.211 with a SD of 0.035. Thus, the cut-off value was determined to be 0.316. However, as the highest P/N value of r*Tm*-p36 was only 1.5 (see Additional file 3: Table S2), we stopped the further exploration of the serodiagnostic potential of *Tm*-p36.

**Fig. 6:**
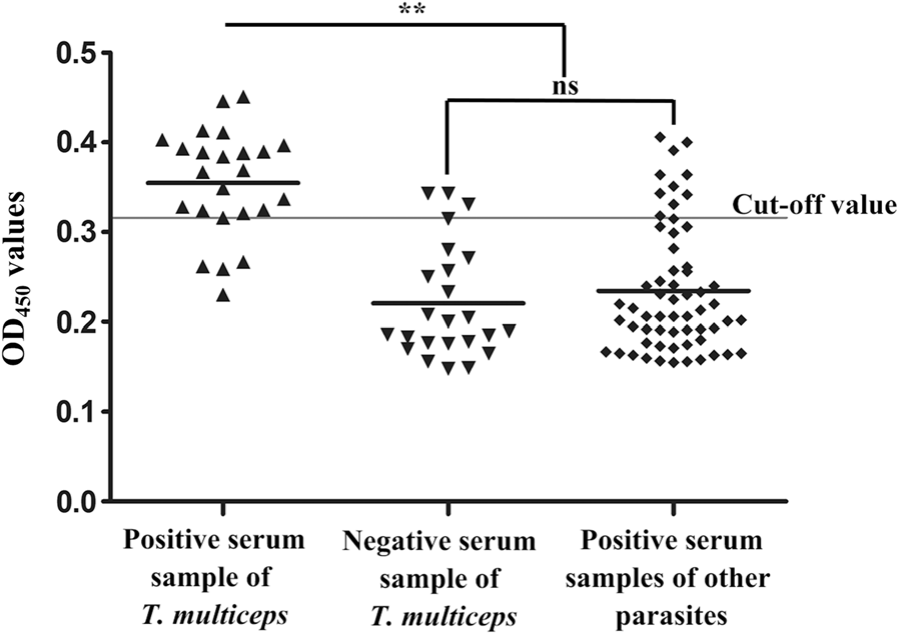
Sensitivity, specificity and cross-reactivity of indirect ELISA based on r*Tm*-HSP60. The bold horizontal line represents the cut-off value (0.316). Statistically significant differences were observed between *T. multiceps*-positive sera and the other serum samples, including *E. granulosus*, *M. expansa*, *T. hydatigena*, *F. hepatica*, *H. contortus*-positive and *T. multiceps*-negative serum samples (Mann-Whitney U-test, *Z* = -5.261, *P* < 0.001). No difference was noted between the *T. multiceps*-negative and other parasites-positive serum samples (Mann-Whitney U-test, *Z* = 0.699, *P =* 0.484)

The sensitivity of indirect ELISA identified by positive serum samples was 83.3% (20/24), and the specificity by negative serum samples was 87.5% (21/24). No cross-reaction between r*Tm*-HSP60 and antibodies of *T. hydatigena* (*n* = 12), *F. hepatica* (*n* = 12) and *H. contortus* (*n* = 12) was obvious, but cross-reactivity was observed with five serum samples of *E. granulosus* (*n* = 12) and five of *M. expansa* (*n* = 12) (Fig. 6).

## Discussion

HSPs are a family of proteins expressed in all prokaryotes and eukaryotes during growth and development, and expression is stimulated by various physical, chemical and pathological factors. These non-secretory proteins are among the most highly conserved among development-related proteins and are also the most important molecular chaperones [22]. The main biological function of HSPs is to facilitate the folding, translocation, renaturation and degradation of their protein substrates in order to protect cells from harmful factors, both internally and externally in all organisms, ranging from mammals to microbes, consistent with their important roles in all species [23].

Traditionally, HSPs can be divided into eight groups according to their molecular weight, including sHSPs that have a molecular weight ranging between 12–43 kDa [24]. Like other HSPs, sHSPs are involved in many cellular processes, and most are characterised by a conserved C-terminal α-crystallin domain of 80–100 residues [25]. They usually form large homo- or heteromeric complexes to achieve chaperone-like and other activities [26].

In parasites, HSPs play an extremely important role in life processes, many of which pass through multiple life-cycle stages, during which parasites must face a variety of environmental challenges such as severe hypoxia, high redox conditions, and temperature changes [27]. Adult worms of *T. multiceps* adhere to the small intestine of their hosts, while metacestodes are present in the central nervous system and subcutaneous or muscular tissues of the intermediate hosts and oncospheres invade intermediate hosts through the intestine; all these processes occur in harsh conditions [28, 29]. In response to these harsh living conditions, parasite HSPs may play an important role. In addition to their molecular chaperone activities, HSPs are also involved in the growth and development of parasites and can be recognised by the host immune system as an important antigen during invasion [30–33].

*Tm*-HSP60 shares high homology with the GroEL molecular chaperone in *Escherichia coli*. The ATP/Mg^2+^ binding site is highly similar to that in HSPs in mammals and bacteria, and these sites are known to be important for molecular chaperone activity [34, 35]. The expression of *Tm*-HSP60 at all life stages indicates that *Tm*-HSP60 possibly plays an important role throughout the growth and development of *T. multiceps*. However, subtle differences were apparent, since *Tm*-HSP60 was mainly distributed in immature stages (scolex, immature proglottid and oncosphere). It may be involved in the processing of target proteins to protect the body at all stages of growth in response to environmental changes. In a previous study, *Tm*-HSP70 was used as an important diagnostic antigen in goats with coenurosis [14]. Similarly, *Tm*-HSP60 also displayed immunogenicity in the present work. Corresponding antibodies were detected from the serum of infected goats, indicated that this protein was recognised by the host as an important antigen during invasion. Therefore, we established an indirect ELISA to assess the serological diagnostic potential of r*Tm*-HSP60. A definite sensitivity (83.3%) and specificity (87.5%) was observed. There was no cross-reaction with other species except for cestodes which is most likely due to high affinity. Non-homologous short fragments containing more antigenic epitopes may improve this situation. Feasible results suggested that *Tm*-HSP60 can be used as a diagnostic tool for coenurosis in the future.

Like metazoan α-crystallin-type sHSPs, *Tm*-p36 has two N-terminal α-crystallin domains, which is essential for sHSPs to perform their chaperone-related functions [36, 37]. As the name suggests, sHSPs have a relatively low molecular weight, but multiple proteins can be assembled into large homo- or heteromeric complexes, consisting of 9–50 subunits with overall molecular weights of 800 kDa or more to form a functional unit [38–40]. The transcriptional level of *Tm*-p36 in adults was low, but higher in protoscolices and oncospheres, especially in the latter, which is consistent with findings in other cestodes [15–18]. The protoscolex is the larval stage of *T. multiceps* (coenurus cerebralis), and the oncosphere is the stage that invades the intermediate host [41]. The specific distribution also indicates that *Tm*-p36 plays an important role in the life-cycle of *T. multiceps*, especially in growth and development of the protoscolices and oncospheres which face harsh living conditions such as extremely high acidity and an anoxic environment in the host immune system. In adult cestodes, oncospheres are also present in the eggs of gravid proglottids, but the results of immunohistochemistry and qPCR analyses revealed low *Tm*-p36 expression level in the gravid proglottids. These results indicate that *Tm*-p36 is closely related to the activation of oncospheres during host invasion. However, *Tm*-p36 does not have good immunoreactivity from the results of western blotting and indirect ELISA. It may reveal that the short-term high expression of *Tm*-p36 is not recognized by the host immune system to produce the corresponding antibody for detection.

Some studies have shown that sHSPs of the parasite is overexpressed at a certain life-cycle stage or under stress, and most of them act as immune system stimulator antigen in host-parasite relationship [42–44]. Previous studies have also suggested that the major egg antigen of *S. mansoni*, also known as *Sm*-p40, is an immune system stimulator associated with granuloma formation inhibition and has been widely studied as a vaccine candidate in schistosomiasis [45–47]. Considering the similar situation, *Tm*-p36 could also serve as an important vaccine or therapeutic target for inhibiting activation or preventing invasion of oncospheres.

## Conclusions

In conclusion, we performed sequence, structure, tissue distribution and transcriptional profile analyses of two HSPs in *T. multiceps* and found that *Tm*-HSP60 is widely distributed in all life-cycle stages, and compared with *Tm*-p36, *Tm*-HSP60 displayed stronger immunogenicity. Thus, *Tm*-HSP60 is of interest for antibody detection, while *Tm*-p36 was most strongly associated with oncospheres and protoscolices, and is of potential interest for vaccination for controlling coenurus cerebralis in the future.

## Additional files

**Additional file 1: Figure S1.** Western blot of r*Tm*-p36 with six coenurus cerebralis-positive sera. Lane M, protein molecular weight markers; Lane C, western blot of r*Tm*-p36 (10 µg) with negative control goat serum.

**Additional file 2: Table S1.** Determination of the optimal r*Tm*-HSP60 coating concentration and serum dilution for indirect ELISA.

**Additional file 3: Table S2.** Determination of the optimal r*Tm*-p36 coating concentration and serum dilution for indirect ELISA.

## Abbreviations

HSPs: heat-shock proteins
sHSPs: small heat-shock proteins
qPCR: quantitative real-time PCR
IPTG: 1 mM isopropyl β-d-1-thiogalactopyranoside
ELISA: enzyme-linked immunosorbent assay
IgG: immunoglobulin G
SDS-PAGE: sodium dodecyl sulphate-polyacrylamide gel electrophoresis
TBST: TRIS-buffered saline containing Tween-20
PBS: phosphate-buffered saline
r*Tm*-HSP60: recombinant *T. multiceps* HSP60
r*Tm*-p36: recombinant *T. multiceps* p36

## References

[1] Xing H, Lin C, Yang Y, Gu X, Yu W, Lai W (2015). Expression, tissue localization and serodiagnostic potential of *Taenia multiceps* acidic ribosomal protein P2. Parasit Vectors..

[2] Li WH, Qu ZG, Zhang NZ, Yue L, Jia WZ, Luo JX (2015). Molecular characterization of enolase gene from *Taenia multiceps*. Res Vet Sci..

[3] Merbl Y, Shilobenjamini Y, Chai O, Chamisha Y, Anglister N, King R (2014). *Taenia multiceps* brain cyst removal in two wild nubian ibex (*Capra nubianas*). J Zoo Wildl Med..

[4] Wang N, Wang Y, Ye Q, Yang Y, Wan J, Guo C (2018). Development of a direct PCR assay to detect *Taenia multiceps* eggs isolated from dog feces. Vet Parasitol..

[5] Xing H, Jing X, Yu W, Cheng G, Lin C, Gu X (2016). GP50 as a promising early diagnostic antigen for *Taenia multiceps* infection in goats by indirect ELISA. Parasit Vectors..

[6] Dehghani M, Mohammadi MA, Rostami S, Shamsaddini S, Mirbadie SR, Harandi MF (2016). High-resolution melting analysis (HRM) for differentiation of four major *Taeniidae* species in dogs *Taenia hydatigena*, *Taenia multiceps*, *Taenia ovis*, and *Echinococcus granulosus**sensu stricto*. Parasitol Res..

[7] Cheng G, Yu W, Xing H, Ning W, Ming Y, Ran H (2017). Molecular cloing and bioinformatics analysis of lactate dehydrogenase from *Taenia multiceps*. Parasitol Res..

[8] Hermos JA, Healy GR, Schultz MG, Barlow J, Church WG (1970). Fatal human cerebral coenurosis. JAMA..

[9] Nie HM, Xie Y, Fu Y, Yang YD, Gu XB, Wang SX (2013). Cloning and characterization of the fatty acid-binding protein gene from the protoscolex of *Taenia multiceps*. Parasitol Res..

[10] Li W, Liu B, Yang Y, Ren Y, Wang S, Liu C (2018). The genome of tapeworm *Taenia multiceps* sheds light on understanding parasitic mechanism and control of coenurosis disease. DNA Res..

[11] Maresca B, Carratù L (1992). The biology of the heat shock response in parasites. Parasitol Today..

[12] Vonlaufen N, Kanzok SM, Wek RC, Sullivan SW (2008). Stress response pathways in protozoan parasites. Cell Microbiol..

[13] Martinez J, Perezserrano J, Bernadina WE, Rodriguezcaabeiro F (1999). *Echinococcus granulosus*: *in vitro* effects of ivermectin and praziquantel on hsp60 and hsp70 levels. Exp Parasitol..

[14] Wang Y, Nie H, Gu X, Wang T, Huang X, Chen L (2015). An ELISA using recombinant *Tm*HSP70 for the diagnosis of *Taenia multiceps* infections in goats. Vet Parasitol..

[15] Kappé G, Aquilina JA, Wunderink L, Kamps B, Robinson CV, Garate T (2004). *Ts*p36, a tapeworm small heat-shock protein with a duplicated alpha-crystallin domain, forms dimers and tetramers with good chaperone-like activity. Proteins..

[16] Santivañez SJ, Hernández-González A, Chile N, Oleaga A, Arana Y, Palma S (2016). Proteomic study of activated *Taenia solium* oncospheres. Mol Biochem Parasitol..

[17] Wang Y, Xiao D, Shen Y, Han X, Zhao F, Li X (2015). Proteomic analysis of the excretory/secretory products and antigenic proteins of *Echinococcus granulosus* adult worms from infected dogs. BMC Vet Res..

[18] Benitez L, Harrison LJS, Parkhouse RME, Garate T (1998). Sequence and preliminary characterisation of a *Taenia saginata* oncosphere gene homologue of the small heat-shock protein family. Parasitol Res..

[19] Wu X, Fu Y, Yang D, Zhang R, Zheng W, Nie H (2012). Detailed transcriptome description of the neglected cestode *Taenia multiceps*. PLoS One.

[20] Huang X, Xu J, Chen L, Wang Y, Gu X, Peng X (2017). Analysis of transcriptome data reveals multifactor constraint on codon usage in *Taenia multiceps*. BMC Genomics..

[21] Crowther JR, Walker JM (2009). The ELISA Guidebook.

[22] De MA (1999). Heat shock proteins: facts, thoughts, and dreams. Shock..

[23] Granel B, Swiader L, Serratrice J, Disdier P, Weiller PJ (2000). Heat shock or “stress proteins”. Rev Med Interne..

[24] Feder ME, Hofmann GE (1999). Heat-shock proteins, molecular chaperones, and the stress response: evolutionary and ecological physiology. Annu Rev Physiol..

[25] de Jong WW, Caspers GJ, Leunissen JA (1998). Genealogy of the alpha-crystallin–small heat-shock protein superfamily. Int J Biol Macromol..

[26] Pérez-Morales D, Espinoza B (2015). The role of small heat shock proteins in parasites. Cell Stress Chaperon..

[27] Polla BS (1991). Heat shock proteins in host-parasite interactions. Immunol Today..

[28] Sun Y, Wang Y, Huang X, Gu X, Lai W, Peng X (2017). Characterization of glutathione S-transferase and its immunodiagnostic potential for detecting *Taenia multiceps*. Vet Parasitol..

[29] Wu X, Fu Y, Yang D, Xie Y, Zhang R, Zheng W (2013). Identification of neglected cestode *Taenia multiceps* microRNAs by illumina sequencing and bioinformatic analysis. BMC Vet Res..

[30] Stewart GR, Young DB (2004). Heat-shock proteins and the host-pathogen interaction during bacterial infection. Curr Opin Immunol..

[31] Van EW, Van DZR, Prakken B (2005). Heat-shock proteins induce T-cell regulation of chronic inflammation. Nat Rev Immunol..

[32] Engman DM, Dragon EA, Donelson JE (1990). Human humoral immunity to hsp70 during *Trypanosoma cruzi* infection. J Immunol..

[33] Hedstrom R, Culpepper J, Schinski V, Agabian N, Newport G (1988). Schistosome heat-shock proteins are immunologically distinct host-like antigens. Mol Biochem Parasitol..

[34] Brocchieri L, Karlin S (2000). Conservation among HSP60 sequences in relation to structure, function, and evolution. Protein Sci..

[35] Goloubinoff P, Gatenby AA, Lorimer GH (1989). GroEL heat-shock proteins promote assembly of foreign prokaryotic ribulose bisphosphate carboxylase oligomers in *Escherichia coli*. Nature..

[36] Montfort RV, Slingsby C, Vierlingt E (2001). Structure and function of the small heat shock protein/alpha-crystallin family of molecular chaperones. Adv Protein Chem..

[37] James M, Crabbe C, Hepburne-Scott HW (2001). Small heat shock proteins (sHSPs) as potential drug targets. Curr Pharm Biotechnol..

[38] Macrae TH (2000). Structure and function of small heat shock/alpha-crystallin proteins: established concepts and emerging ideas. Cell Mol Life Sci..

[39] Haslbeck M (2002). sHsps and their role in the chaperone network. Cell Mol Life Sci..

[40] Sun Y, Macrae TH (2005). Small heat shock proteins: molecular structure and chaperone function. Cell Mol Life Sci..

[41] Oryan A, Moazeni M, Amrabadi O, Akbari M, Sharifiyazdi H (2015). Comparison of distribution pattern, pathogenesis and molecular characteristics of larval stages of *Taenia multiceps* in sheep and goats. Small Ruminant Res..

[42] Ferrer E, González LM, Foster-Cuevas M, Cortéz MM, Dávila I, Rodríguez M (2005). *Taenia solium*: characterization of a small heat shock protein (*Ts*ol-sHSP35.6) and its possible relevance to the diagnosis and pathogenesis of neurocysticercosis. Exp Parasitol..

[43] Moxon JV, Lacourse EJ, Wright HA, Perally S, Prescott MC, Gillard JL (2010). Proteomic analysis of embryonic *Fasciola hepatica*: characterization and antigenic potential of a developmentally regulated heat shock protein. Vet Parasitol..

[44] Younis AE, Geisinger F, AjoninaEkoti I, Soblik H, Steen H, Mitreva M (2011). Stage-specific excretory/secretory small heat shock proteins from the parasitic nematode *Strongyloides ratti*: putative links to host’ s intestinal mucosal defense system. FEBS J..

[45] Cai Y, Langley JG, Smith DI, Boros DL (1996). A cloned major *Schistosoma mansoni* egg antigen with homologies to small heat shock proteins elicits Th1 responsiveness. Infect Immun..

[46] Abouel-Nour MF, Lotfy M, Attallah AM, Doughty BL (2006). *Schistosoma mansoni* major egg antigen *Sm*p40: molecular modeling and potential immunoreactivity for anti-pathology vaccine development. Mem Inst Oswaldo Cruz..

[47] Stadecker MJ, Hernandez HJ (2010). The immune response and immunopathology in infection with *Schistosoma mansoni*: a key role of major egg antigen *Sm*-p40. Parasite Immunol..

