# Supplementary figures and images for: Molecular characterisation and expression analysis of two heat-shock proteins in Taenia multiceps
Source: Parasit Vectors. 2019 Mar 12;12:93. doi: 10.1186/s13071-019-3352-8 (PMC6417115; doi:10.1186/s13071-019-3352-8)

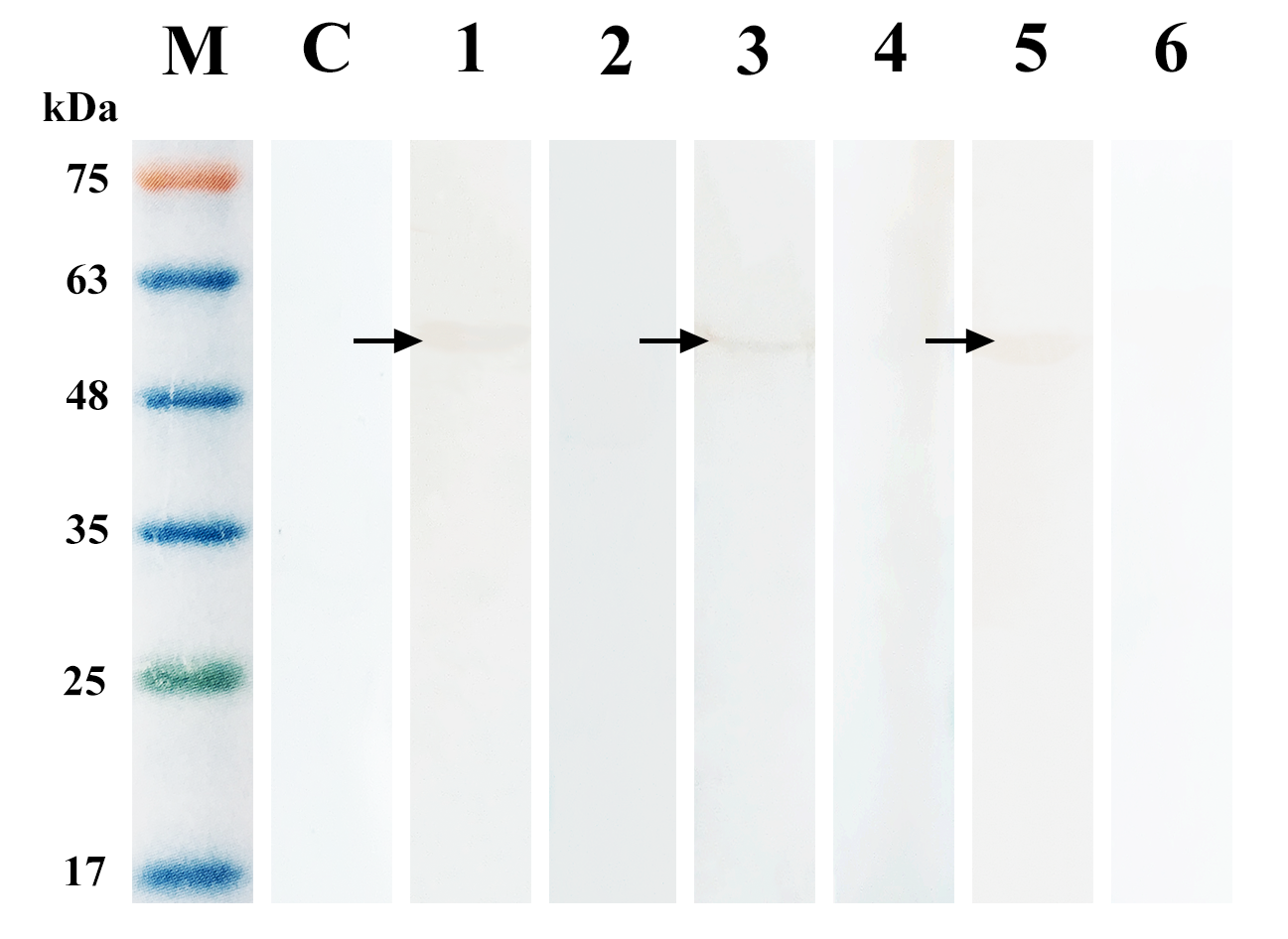

Supplement: Supplementary file 1 — Additional file 1: Figure S1. Western blot of rTm-p36 with six coenurus cerebralis-positive sera. Lane M, protein molecular weight markers; Lane C, western blot of rTm-p36 (10 µg) with negative control goat serum. [file 13071_2019_3352_MOESM1_ESM.tif]
