# Supplementary material for: Molecular characterisation and expression analysis of two heat-shock proteins in Taenia multiceps
Source: Parasit Vectors. 2019 Mar 12;12:93. doi: 10.1186/s13071-019-3352-8 (PMC6417115; doi:10.1186/s13071-019-3352-8)
Supplement: Supplementary file 2 — Additional file 2: Table S1. Determination of the optimal rTm-HSP60 coating concentration and serum dilution for indirect ELISA. [file 13071_2019_3352_MOESM2_ESM.pdf]

Table S1 Determination of the optimal r*Tm*-HSP60 coating concentration and serum dilution for indirect ELISA

Serum dilutions	OD450 values of r*Tm*-HSP60 at different coating concentrations				
	0.1μg	0.2μg	0.4μg	0.8μg	1.6μg
1:20 (P)	0.797	0.835	0.855	0.874	0.918
1:20 (N)	0.598	0.616	0.666	0.677	0.757
1:40 (P)	0.698	0.712	0.723	0.762	0.796
1:40 (N)	0.382	0.452	0.487	0.481	0.583
1:80 (P)	0.543	0.569	0.577	0.585	0.593
1:80 (N)	0.264	0.33	0.358	0.36	0.428
1:160 (P)	0.403	**0.478**	0.482	0.49	0.5
1:160 (N)	0.204	**0.212**	0.268	0.266	0.327
1:360 (P)	0.309	0.312	0.342	0.355	0.389
1:360 (N)	0.15	0.155	0.198	0.198	0.268

Abbreviations: N positive serum, P negative serum

Note: Bold represent the optimum conditions for this indirect ELISA method, the highest P/N value is 2.255
