# Supplementary material for: Molecular characterisation and expression analysis of two heat-shock proteins in Taenia multiceps
Source: Parasit Vectors. 2019 Mar 12;12:93. doi: 10.1186/s13071-019-3352-8 (PMC6417115; doi:10.1186/s13071-019-3352-8)
Supplement: Supplementary file 3 — Additional file 3: Table S2. Determination of the optimal rTm-p36 coating concentration and serum dilution for indirect ELISA. [file 13071_2019_3352_MOESM3_ESM.pdf]

Table S2 Determination of the optimal r*Tm*-p36 coating concentration and serum dilution for indirect ELISA

Serum dilutions	OD450 values of r*Tm*-p36 at different coating concentrations				
	0.6μg	1.2μg	2.4μg	4.8μg	9.6μg
1:20 (P)	1.088	1.189	1.254	1.293	1.327
1:20 (N)	1	1.167	1.243	1.29	1.309
1:40 (P)	0.895	1.073	1.178	1.235	1.298
1:40 (N)	0.803	0.974	1.143	1.103	1.287
1:80 (P)	0.719	0.883	1.003	1.14	1.255
1:80 (N)	0.609	0.801	0.889	0.909	1.098
1:160 (P)	0.533	0.672	0.83	0.95	1.113
1:160 (N)	0.444	0.633	0.698	0.708	0.91
1:360 (P)	0.379	0.475	0.625	**0.758**	0.925
1:360 (N)	0.371	0.452	0.517	**0.505**	0.705
1:640 (P)	0.233	0.271	0.341	0.447	0.556
1:640 (N)	0.203	0.251	0.316	0.359	0.372

Abbreviations: N positive serum, P negative serum

Note: Bold represent the optimum conditions for this indirect ELISA method, the highest P/N value is 1.50.
